# Anticancer drug-induced nephrotoxicity: biopsy-proven patterns and outcomes across chemotherapy, targeted therapy, and immune checkpoint inhibitors

**DOI:** 10.1080/0886022X.2025.2590283

**Published:** 2025-11-25

**Authors:** Jing Tian, Jingying Lian, Yuanyuan Xia, Lei Ma, Mingchao Zhang, Xiaodong Zhu, Gen Wang, Yongzhong Zhong, Caihong Zeng

**Affiliations:** ^a^ National Clinical Research Center for Kidney Diseases, Jinling Hospital, Affiliated Hospital of Medical School, Nanjing University, Nanjing, China; ^b^ National Clinical Research Center for Kidney Diseases, Jinling Hospital, Nanjing Medical University, Nanjing, China; ^c^ Department of Nephrology, Hangzhou TCM Hospital Affiliated to Zhejiang Chinese Medical University, Hangzhou, China

**Keywords:** Cisplatin nephrotoxicity, gemcitabine-associated thrombotic microangiopathy, VEGF inhibitor kidney injury, immune checkpoint inhibitor acute kidney injury, onconephrology biopsy

## Abstract

Biopsy-confirmed anticancer drug-induced kidney injury is underreported. This study aimed to characterize its clinicopathological features and outcomes. We retrospectively analyzed 52 patients with biopsy-proven anticancer drug-induced nephrotoxicity (2005–2024). Patients were classified into chemotherapy drugs (CTD, *n* = 25), molecularly targeted therapies (MTT, *n* = 22), and immune checkpoint inhibitors (ICI, *n* = 5; PD-1 inhibitors). The CTD group (e.g. cisplatin, capecitabine, gemcitabine) caused frequent acute kidney injury (AKI, 80%), with acute tubulointerstitial nephritis (ATIN, 32%). Notable glomerular lesions in the CTD group included thrombotic microangiopathy (TMA, 12%), minimal change disease (8%), and focal segmental glomerulosclerosis (8%). The MTT group (e.g. bevacizumab, lenvatinib, sorafenib) had higher proteinuria (0.4 *vs.* 3.1 *vs.* 0.7 g/24h; *p* < 0.05) and TMA incidence (86%). MTT-induced TMA produced distinct subtypes: anti-VEGF(R) therapy (*n* = 11) caused glomerular capillary ballooning (100%); non-VEGFR-TKIs (*n* = 4) were associated with segmental glomerulopathy; combined anti-VEGF/VEGFR-TKI (*n* = 4) resulted in more extensive and severe TMA (>75% of glomeruli). ICI therapy (nivolumab, camrelizumab, sintilimab) led to early AKI, mainly ATIN (80%), with glomerular IgA deposition (80%) and low serum C3 (60%). After a median follow-up of 23.0 months, MTT showed faster AKI recovery than CTD (0.5 *vs.* 8.0 months; *p* = 0.002). Anticancer drugs induce distinct nephrotoxic patterns. CTD causes direct cytotoxicity and high irreversible injury risk. MTT drives functional TMA, and new-onset hypertension with proteinuria should raise concern for anti-VEGF–related TMA. ICI triggers immune dysregulation with humoral disturbances, and AKI with low serum C3 can be a safety signal for clinical monitoring.

## KEY POINTS

**Anticancer drugs induce distinct nephrotoxic patterns:** chemotherapy drugs (CTD) are associated with direct cellular toxicity and a high risk of irreversible kidney injury; molecularly targeted therapies (MTT) drive functional thrombotic microangiopathy; immune checkpoint inhibitors (ICI) may trigger immune dysregulation.

**Biopsy Guides Management:** Renal biopsy is essential for accurate diagnosis, especially in MTT-related injury, where pathological heterogeneity correlates with drug targets and affects treatment approaches.

**Prognostic Heterogeneity:** Injury from MTT usually resolves quickly after drug discontinuation, while CTD-related injury carries a higher risk of irreversible damage. ICI-related injury typically responds to steroids but may have a poorer outcome if glomerular pathology is present.

**Novel Immune Mechanisms:** ICI-associated injury frequently involves glomerular IgA deposition and complement activation, suggesting that humoral immunity may play a role beyond classic T-cell-mediated inflammation.

**Framework for Onconephrology:** This large biopsy-based study helps clinicians optimize management across three anticancer drug classes.

## Graphical Abstract

**Figure:**
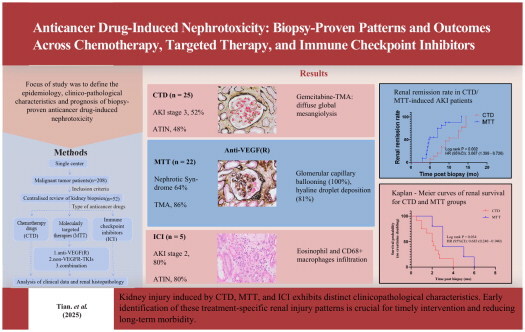

## Introduction

The use of anticancer drugs has significantly improved survival rates in cancer patients. However, their nephrotoxicity poses substantial challenges to the efficacy and long-term sustainability of cancer treatment. Despite clinical data on renal adverse events of the chemotherapy drugs (CTDs) existing [1,2], systematic pathological research based on renal biopsy remains limited, particularly for newer therapies, such as molecular targeted drugs (MTTs) and immune checkpoint inhibitors (ICIs). Kidney injuries associated with these emerging therapies demonstrate considerable clinical and histopathological heterogeneity [3–5]. However, current understanding relies heavily on isolated case reports, with minimal comparative analysis across drug categories or mechanistic exploration of target-specific injury patterns. In particular, the pathological heterogeneity of molecular target-associated glomerular diseases remains poorly characterized. Therefore, this study has addressed these gaps. To our knowledge, this is the largest biopsy-based cohort that directly compares clinicopathological patterns and prognostic outcomes across the three major anticancer drug classes (CTD, MTT, and ICI). This work may enhance our understanding and provide novel insights into the nephrotoxicity of anticancer drugs.

## Materials and methods

### Patients

Patients with biopsy-confirmed anticancer drug-induced nephrotoxicity from February 2005 to August 2024 were included. The inclusion criteria were as follows: (1) age ≥18 years; (2) histologically confirmed malignancy with normal baseline renal function (serum creatinine <1.2 mg/dL) and urinalysis (proteinuria <150 mg/24h) before anticancer therapy; (3) renal insufficiency or abnormal urinalysis following systemic therapy with CTD, MTT, or ICI within 1 year before renal biopsy, with the kidney injury attributed to anticancer drug therapy based on clinical evaluation and biopsy results; (4) regular blood biochemical tests and urinalysis conducted during and after anticancer therapy. Exclusion Criteria included: (1) history of chronic kidney disease; (2) transplanted kidneys; (3) pre-kidney, post-kidney injury or direct infiltration of cancer cells into the kidney; (4) incomplete or missing data.

Patients were categorized into three groups according to the type of anticancer drugs received within one year before renal biopsy: the CTD, MTT, or ICI groups. Cases with multidrug exposure were stratified by nephrologists and pathologists according to clinicopathological correlations and therapeutic withdrawal responses. CTD group: patients administered ≥1 traditional cytotoxic anticancer drugs, including platinum derivatives (cisplatin, oxaliplatin, carboplatin, nedaplatin), antimetabolites (fluorouracil, capecitabine, tegafur), taxanes (paclitaxel, docetaxel), anthracyclines (epirubicin), alkylating agents (fotemustine), antifolates (pemetrexed), topoisomerase inhibitors (etoposide), nucleoside analogs (gemcitabine), and non-cytotoxic endocrine therapy (bicalutamide, goserelin). MTT group: patients receiving molecular targeted therapies, including anti-VEGF(R) drugs (bevacizumab, lenvatinib, anlotinib, apatinib, sorafenib, fruquintinib, sunitinib), breakpoint cluster region-Abelson (BCR-ABL) inhibitors (dasatinib, nilotinib, imatinib), and EGFR inhibitors (almonertinib, cetuximab), either as monotherapy or in combination therapy. ICI group: patients administered ICIs, specifically PD-1 inhibitors (camrelizumab, nivolumab, and sintilimab), either as monotherapy or combined with CTD/MTT. The study has been reviewed and approved by the Jinling Hospital Ethics Committee (reference number: 2024DZKY-083-01). Informed written consent was obtained from all individual participants included in the study.

### Clinical evaluation

Data were collected from medical records, and changes in treatment medications, proteinuria, and renal function were monitored during the follow-up period. Acute kidney injury (AKI) was staged according to the KDIGO 2012 criteria [6]. Estimated glomerular filtration rate (eGFR) was calculated using the CKD-EPI formula. Renal response was defined as follows: complete recovery (CR): SCr <1.24 mg/dL and 24-h urine protein <0.4 g/d; partial recovery (PR): SCr ≥25% reduction in SCr from the peak concentration but still above 1.24 mg/dL, or >50% decrease in 24-h urine protein from baseline values and 24-h urine protein between 0.4 and 3.5 g/d; non-recovery (NR): failure to achieve CR or PR despite clinical therapy. The follow-up endpoint was defined as the combined endpoint of SCr <1.24 mg/dL and 24-h urine protein <0.4 g/d. Cumulative renal remission rate: *a* ≥ 25% decrease in SCr from the peak concentration or *a* > 50% decrease in 24-h urine protein from baseline. Doubling of serum creatinine level was defined as a serum creatinine value that increased to at least double the baseline value and was above an upper reference limit of 1.24 mg/dL, with persistent maintenance of the abnormal level throughout the subsequent follow-up period without regression to the normal value. Serum complement C3 and C4 levels were measured, with normal ranges defined as 0.8–1.8 g/L for C3 and 0.10–0.40 g/L for C4. End-stage kidney disease (ESKD) was defined as eGFR <15 mL/min/1.73 m^2^ or the need for renal replacement therapy for more than 3 months.

### Renal histopathology

All renal biopsies were processed using the standard techniques of LM, IF, and EM. Light microscopic tissues were paraffin-embedded, sectioned at 3 μm, and stained with hematoxylin-eosin (HE), periodic acid-Schiff (PAS), periodic acid-silver methenamine (PASM)-Masson, and Masson trichrome. Immunofluorescence frozen tissue sections of 3 μm were stained with IgG, IgA, IgM, C3, and C1q (direct method, Dako). Immunofluorescence staining intensity was graded on a scale of 0 to +++. On light microscope, acute tubular injury and tubular atrophy and interstitial fibrosis were graded on a semiquantitative scale based on an estimate of the percentage of renal cortex affected and recorded as score 0 (<10%), score 1 (10%–25%), score 2 (26%–50%), and score 3 (>50%). Ultrastructural changes were examined using a transmission electron microscope. The severity of the glomerular basement membrane (GBM) double contours was categorized as focal (<50%) or diffuse (≥50%) based on the cumulative glomerular vascular loop area. The glomerular subendothelial width was measured as the vertical distance from the endothelial cell border to the dense layer of the basement membrane. For each case, at least three images were analyzed, with ten measurements taken per average image.

### Statistical analysis

Statistical analysis was performed using SPSS 25.0 (IBM SPSS Statistics for Windows, IBM Corp, Armonk, NY). Normally distributed data are presented as mean ± standard deviation and analyzed by one-way ANOVA, with post-hoc LSD tests for multiple comparisons. Non-normally distributed continuous variables are expressed as medians (interquartile range) and compared using the Kruskal-Wallis test with Bonferroni correction. Categorical variables are described as percentages and analyzed using Fisher’s exact test with Bonferroni adjustment. Kaplan-Meier survival curves were constructed to evaluate survival rates, and group comparisons were performed using the log-rank test. Statistical significance was assumed at *p* < 0.05.

## Results

### Patient selection, baseline demographic and clinical characteristics of study cohort

Between February 2005 and August 2024, 208 patients with malignancies who received oncological treatment prior to renal biopsy were evaluated at the National Clinical Research Center for Kidney Diseases, Jinling Hospital. After applying standardized diagnostic criteria, 52 patients were included in the final analysis. The detailed clinicopathological characteristics and outcomes for all 52 patients are provided in the supplementary dataset available on Zenodo [DOI: 10.5281/zenodo 17225483]. The most common malignancy was gastrointestinal tumors (23 cases, 44%), followed by lung tumors (14 cases, 27%), liver tumors (5 cases, 9%), chronic myelogenous leukemia (4 cases, 8%), gynecological tumors (3 cases, 6%), urinary tract tumors (1 case, 2%), thymoma (1 case, 2%), and thyroid tumors (1 case, 2%) (Table 2). Overall, 52 patients were categorized as follows: 25 cases (48%) received CTD, 22 cases (42%) received MTT, and 5 cases (10%) received ICI. In the CTD group, cisplatin was the most frequently administered (7/25 cases, 28%), followed by capecitabine (6 cases, 24%), oxaliplatin, paclitaxel, and docetaxel (4 cases each, 16%). The MTT group was predominantly treated with bevacizumab (9/22 cases, 41%), followed by anlotinib (3 cases, 13%) and lenvatinib (2 cases, 9%). The ICI group included camrelizumab (2/5 cases, 40%), nivolumab (2 cases, 40%), and sintilimab (1 case, 20%) (Table 1). Tumor resection had been performed in 75% of patients (*n* = 33), and 27% (*n* = 12) received radiotherapy. Complete tumor remission was most commonly observed in the CTD cohort, whereas metastasis rates and advanced tumor stages were more prevalent in the MTT group. Additionally, the utilization of proton pump inhibitors was more frequent in the CTD group (CTD: 60% *vs.* MTT:18% *vs.* ICIs: 20%; *p* < 0.05), whereas diuretics were more frequently prescribed in the MTT group (CTD: 12% *vs.* MTT:41% *vs.* ICIs: 20%; *p* < 0.05). No significant differences were observed among groups regarding tumor resection rates or comorbidities (Table 2).

**Table 1.** Anticancer drugs composition in the study population.

CTD (*n* = 25)		MTT (*n* = 22)		ICI (*n* = 5)	
Categories	n(%)	Categories	n(%)	Categories	n(%)
Platinum-based	15 (60)	Anti-VEGF(R)	16 (73)	PD-1 Inhibitors	5 (100)
Cisplatin	7 (28)	Bevacizumab	6 (27)	Camrelizumab	2 (40)
Oxaliplatin	4 (16)	Bevacizumab + anlotinib	3 (13)	Sintilimab	2 (40)
Carboplatin	3 (12)	Lenvatinib	2 (9)	Nivolumab	1 (20)
Nedaplatin	1 (4)	Apatinib	2 (9)	Dual therapy^##^	1 (20)
Fluoropyrimidines	9 (36)	Sorafenib	1 (5)	Triple therapy^###^	4 (80)
Capecitabine	6 (24)	Fruquintinib	1 (5)		
Fluorouracil	2 (8)	Sunitinib	1 (5)		
Tegafur	1 (4)	Anti-BCR-ABL	4 (19)		
Taxanes	9 (36)	Dasatinib	2 (9)		
Paclitaxel	5 (20)	Nilotinib	1 (5)		
Docetaxel	4 (16)	Imatinib	1 (5)		
Others	10 (40)	Anti-EGFR	2 (10)		
Gemcitabine	3 (12)	Aumolertinib	1 (5)		
Pemetrexed	2 (8)	Cetuximab	1 (5)		
Fotemustine	1 (4)	Dual therapy^#^	13 (59)		
Etoposide	1 (4)				
Epirubicin	1 (4)				
Bicalutamide	1 (4)				
Goserelin	1 (4)				
Dual therapy^#^	1 (4)				

CTD: chemotherapy drugs; MTT: molecularly targeted therapies; ICI: immune checkpoint inhibitors; Anti-VEGF(R): VEGF/R inhibitors, including anti-VEGF antibody and VEGFR tyrosine kinase inhibitors; Anti-BCR-ABL: Anti-BCR-ABL tyrosine kinase antibody; Anti-EGFR: Anti-epidermal growth factor receptor tyrosine kinase (EGFR) antibody; PD-1 inhibitors: programmed cell death protein-1 inhibitors. Dual therapy#: CTD combined with MTT; Dual therapy##: ICI combined with MTT; Triple therapy###: ICI combined with CTD and MTT.

**Table 2.** Cancer category and treatment-related information in patients with anticancer drug-induced nephrotoxicity.

Baseline variable	Overall (*n* = 52)	CTD (*n* = 25)	MTT (*n* = 22)	ICI (*n* = 5)	*P* value
Cancer category, n (%)					
Gastrointestinal Tumors	23 (44)	12 (48)	8 (36)	3 (60)	0.462
Lung Tumors	14 (27)	8 (32)	6 (27)	0	0.368
Urinary Tract Tumors	1 (2)	1 (4)	0	0	0.444
Liver Tumors	5 (9)	0	3 (13)	2 (40)	0.052
Gynecological Tumors	3 (6)	3 (12)	0	0	0.078
Thymoma	1 (2)	1 (4)	0	0	0.444
Thyroid Tumors	1 (2)	0	1 (4)	0	0.6
Chronic Myelogenous Leukemia	4 (8)	0	4 (18)	0	0.111
Tumor Resection, n (%)	33 (63)	15 (60)	15 (68)	3 (60)	0.267
Previous Radiotherapy, n (%)	12 (23)	3 (12)^a^	6 (27)^c^	3 (60)	0.025
Cancer Status, n (%)					
Complete Remission	15 (29)	13 (52)^a,b^	1 (4)	1 (20)	0.007
Partial Remission	8 (15)	5 (20)	1 (4)	2 (40)	0.079
Stable	19 (37)	5 (20)	12 (54)	2 (40)	0.288
Progression	10 (19)	2 (8)	8 (36)	0	0.084
Metastasis, n (%)	20 (38)	3 (12)^a^	14 (63)	3(60)	0.003
Comorbidities, n (%)	35 (67)	12 (48)	19 (86)	4 (80)	0.280
Diabetes	12 (23)	3 (12)	8 (36)	1 (20)	0.351
Hypertension	16 (31)	5 (20)	9 (41)	2 (40)	0.828
Cardiovascular Diseases	7 (13)	4 (16)	2 (9)	1 (20)	0.279
Concomitant Medications, n (%)					
PPIs	20 (38)	15 (60)^a,b^	4 (18)	1 (20)	0.036
NSAIDs	11 (21)	6 (24)	5 (23)	0	0.495
Antibiotics	12 (23)	8 (32)	2 (9)	2 (40)	0.086
Diuretics	13 (25)	3 (12)^a^	9 (41)^c^	1 (20)	0.041

CTD: chemotherapy drugs; MTT: molecularly targeted therapies; ICI: immune checkpoint inhibitors; BMI: body mass index; PPIs: proton pump inhibitors; NSAIDs: nonsteroidal anti-inflammatory drugs; AKI: acute kidney injury; a: CTD *vs.* MTT; b: CTD *vs.* ICI; c: MTT *vs.* ICI.

At the time of biopsy, the patients had a mean age of 56 ± 10 years, and the male-to-female ratio was 3:1. The median interval from the initiation of anticancer treatment to renal biopsy was 4.5 months (2.0–10.0 months). Comparative analysis revealed significant differences in kidney injury patterns among the three treatment groups. The ICI group demonstrated the shortest median interval from therapy initiation to renal biopsy at 2.0 months (1.0–4.0 months), while the MTT group showed the longest at 7.5 months (2.7–10.5 months). The CTD group displayed the highest median SCr levels (CTD: 3.2 *vs.* MTT: 1.0 *vs.* ICI: 2.2 mg/dL; *p* < 0.01) and a high incidence of AKI (80%), with stage 3 AKI predominating (52%). In contrast, the MTT group exhibited the most severe proteinuria (CTD: 0.4 *vs.* MTT: 3.1 *vs.* ICI: 0.7 g/24h; *p* < 0.05) and the lowest serum albumin levels (CTD: 38.5 *vs.* MTT: 31.8 *vs.* ICI: 36.7 g/L; *p* < 0.05), while showing minimal elevation in SCr and renal tubular injury markers. All patients in the ICI group developed AKI, predominantly stage 2 (80%), with minimal or absent proteinuria (Table 3). Critically, 3 of 5 patients (60%) exhibited significantly reduced serum complement C3 levels (Table 6).

**Table 3.** Baseline clinical characteristics of the different groups at the time of renal biopsy.

Variables	Overall (*n* = 52)	CTD (*n* = 25)	MTT (*n* = 22)	ICI (*n* = 5)	*P* value
Age, years	56 ± 10	56 ± 12	57 ± 8	57 ± 7	0.912
Male, n (%)	39 (75)	19 (76)	15 (68)	5 (100)	0.345
BMI, kg/m^2^	23.8 ± 3.2	23.5 ± 2.4	24.2 ± 3.1	24.6 ± 5.9	0.945
Smoking, n (%)	21 (40)	9 (36)	8 (36)	4 (80)	0.15
Time from Tumor Treatment to Renal Biopsy, months	4.5 (2.0–10.0)	5.0 (2.0–10.5)	7.5 (2.7–10.5)	2.0 (1.0–4.0)	0.18
Serum Creatinine, mg/dL	1.6 (0.9–3.2)	3.2 (1.4–6.3)^a^	1.0 (0.7–1.6)	2.2 (1.4–2.6)	0.003
eGFR, mL/min·1.73 m²	37.0 (18.0–72.0)	27.0 (9.0–59.0)^a^	66.5 (25.5–87.8)	33.0 (20.5–65.0)	0.045
Serum Albumin, g/L	35.5 (29.2–40.5)	38.5 (35.4–42.6)^a^	31.8 (27.3–37.7)	36.7 (25.6–39.8)	0.023
Hemoglobin, g/dL	100.0 (85.0–115.5)	100.0 (76.7–108.0)	112.0 (93.0–141.0)	77.0 (71.0–91.0)	0.078
LDH, U/L	257.0 (180.0–297.7)	260.0 (183.5–391.7)	258.0 (174.0–300.5)	202.0 (121.0–360.5)	0.916
C3, g/L	0.9 (0.2–1.2)	0.8 (0.4–1.3)	1.2 (0.7–1.2)	0.2 (0.2–0.9)	0.195
C4, g/L	0.3 (0.2–0.7)	0.3 (0.2–0.4)	0.3 (0.1–0.8)	0.4 (0.1–0.9)	0.91
Proteinuria, g/24h	1.5 (0.5–3.5)	0.4 (0.2–1.14)^a^	3.1 (1.6–3.9)	0.7 (0.3–1.5)	0.025
Microscopic Hematuria, /μl	13.4 (2.6–41.0)	13.3 (8.5–81.6)	16.8 (10.3–98.1)	2.0 (1.4–5.9)	0.231
uNGAL, ng/mL	65.0 (50.0–230.0)	84.0 (30.0–153.2)^a^	60.0 (35.0–88.1)^c^	368.0 (63.5–1469.0)	0.046
AKI, n (%)	34 (65)	20 (80)^a^	9 (40)^c^	5 (100)	0.004
AKI Stage 1	10 (19)	4 (16)	6 (27)	0	0.42
AKI Stage 2	8 (15)	3 (12)^a^	1 (5)^c^	4 (80)	<0.001
AKI Stage 3	16 (31)	13 (52)^a^	2 (9)^c^	1 (20)	0.016

CTD: chemotherapy drugs; MTT: molecularly targeted therapies; ICI: immune checkpoint inhibitors; BMI: body mass index; eGFR: estimated glomerular filtration rate; LDH: lactate dehydrogenase; C3: complement C3; C4: complement C4; uNGAL: urinary neutrophil gelatinase-associated lipocalin; AKI: acute kidney injury; a: CTD *vs.* MTT; b: CTD *vs.* ICI; c: MTT *vs.* ICI.

**Table 6.** Clinicopathological characteristics of 5 cases of ICI-induced nephrotoxicity.

No.	1	2	3	4	5
Age, years	61	56	45	65	56
Sex	Male	Male	Male	Male	Male
ICI	Sintilimab	Camrelizumab	Nivolumab	Sintilimab	Camrelizumab
Treatment Cycles, cycles	3	2	3	2	1
Interval between ICI and AKI, months	2	4	4	1	1
SCr at Kidney Biopsy, mg/dL	2.73	1.98	2.32	2.45	2.2
AKI Stage	2	2	2	3	2
C3 (g/L)	0.64↓	0.98	0.76↓	1.22	0.75↓
C4 (g/L)	0.20	0.23	0.17	0.15	0.32
Extra-Renal Manifestations	Interstitial pneumonia with interstitial fibrosis, hepatitis, hypothyroidism	Interstitial pneumonia, hypothyroidism	Hepatitis, hypothyroidism	Hepatitis	Hypothyroidism
Pattern of kidney injury	ATIN+IgAN	ATIN	ATIN	ACTIN	ATIN+IgAN
Globally sclerosed glomeruli (%)	7	6	5	22	55
Immunofluorescence Microscopy (CW or mesangial)	C3 (2+), IgA (2+), C1q (1+)	IgA (2+), IgM (1+)	IgA (1+)	C3 (2+)	C3 (1+), IgA (2+)
Tubulitis	＋	－	＋	＋	＋
Treatment	Methylprednisolone 20 mg/d	Methylprednisolone 200 mg/d	Methylprednisolone 30 mg/d	Prednisone 30 mg/d + CRRT	Methylprednisolone 60 mg/d
Renal Prognosis	Dead	CR	PR	PR	NR

ICI: immune checkpoint inhibitors; AKI: acute kidney injury; SCr: serum creatinine; ATIN: acute tubulointerstitial nephritis; IgAN: immunoglobulin A nephropathy; ACTIN: acute combined chronic tubulointerstitial nephritis; CW: glomerular capillary wall; IgA: immunoglobulin A; IgM: immunoglobulin M; C3: complement C3; C4: complement C4; C1q: complement C1q; Immunohistochemistry: assess the extent of interstitial infiltrating cells based on the original pathological images: 2 +, strong; 1+, moderate. CRRT: continuous renal replacement therapy; CR: complete recovery of blood creatinine; PR: partial recovery of blood creatinine; NR: non-remission is the failure to achieve CR or PR despite clinical therapy.

### Pathologic characteristics

**Figure 1.**
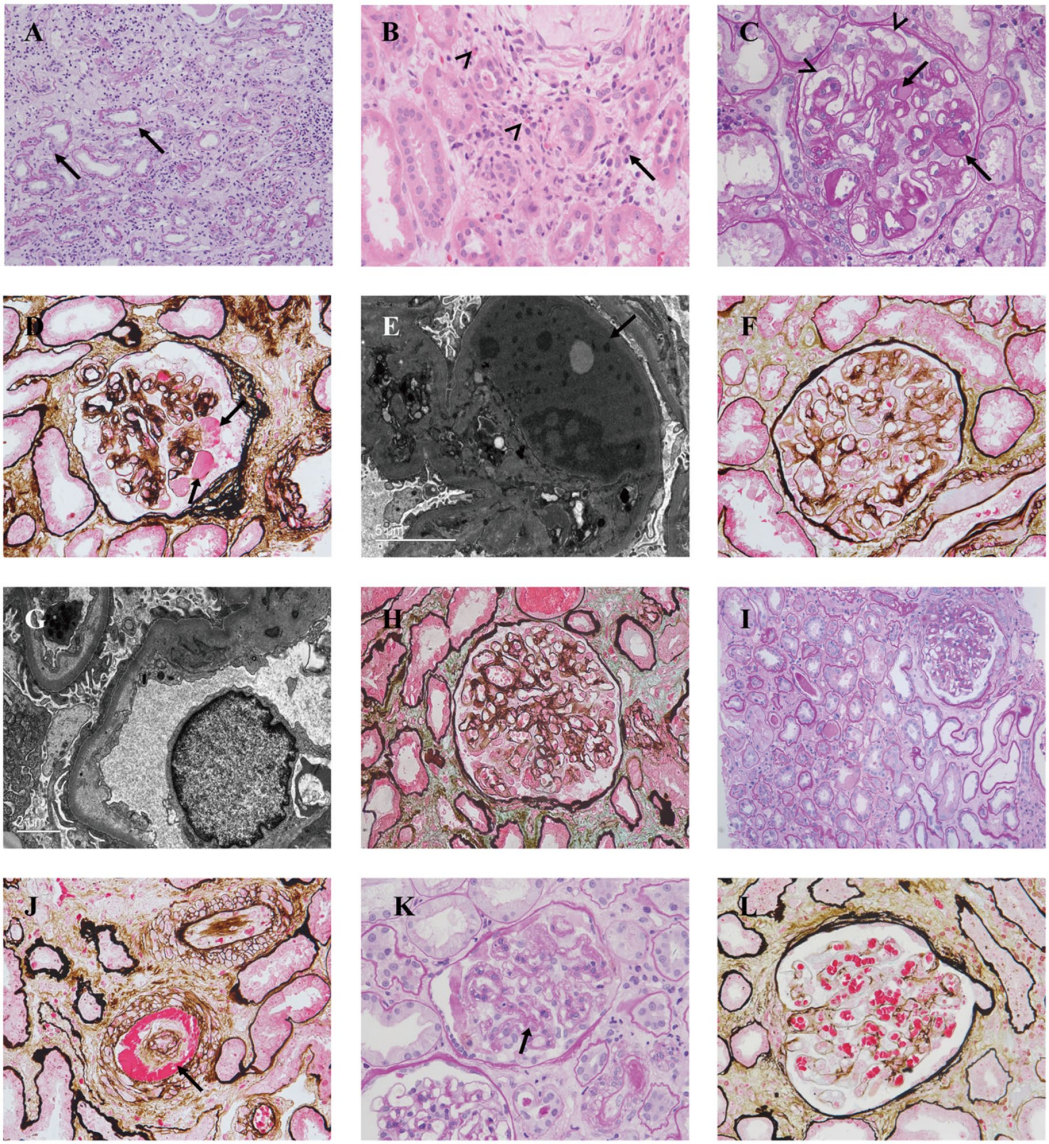
Pathological features of kidney injury in patients treated with anticancer drugs. (A-B) ICI-related ATIN: tubulointerstitial damage with extensive infiltration of mononuclear cells, eosinophils (arrowhead), plasma cells (arrows), and neutrophils, accompanied by tubulitis (arrows). (C-E) Bevacizumab-associated TMA: C-D Severe glomerular endothelial cell injury with patent capillary lumens and multiple peripheral aneurysmal dilations (arrow head); PAS-positive or weakly positive subendothelial and intraluminal deposits (arrows); E Hyaline droplet-like material in the subendothelial region (arrows). (F-G) BCR-ABL (Dasatinib)-associated TMA: mild lesion characterized by segmental mesangiolysis and double-track signs. Under electron microscopy, podocyte foot process effacement was observed. (H-I) EGFR (Cetuximab)-associated TMA: mild lesions primarily involving endothelial injury, with segmental endothelial hyperplasia and double-track signs, accompanied by moderate acute tubular-interstitial damage and significant interstitial inflammation. (J) BCR-ABL (Imatinib)-associated TMA: vascular TMA lesions are characterized by the presence of fibrinoid material and red blood cell fragments within the vessel walls, accompanied by intimal edema (arrows). (K-L) Gemcitabine-associated TMA: K Prominent double-track signs (arrows); L Diffuse global mesangiolysis and red blood cell aggregation.

Comparative analysis of renal biopsy revealed distinct histopathological patterns associated with different classes of anticancer drugs (Table 4). In the CTD group, acute tubulointerstitial nephritis (ATIN; acute interstitial nephritis, AIN) was observed in 32% of cases. Notable glomerular lesions were also observed, including thrombotic microangiopathy (TMA) (3 cases, 12%), minimal change disease (MCD) (2 cases, 8%), and focal segmental glomerulosclerosis (FSGS) (2 cases, 8%). Three cases of gemcitabine-induced TMA presented with the most severe and aggressive clinical manifestations, including hypertension, anemia, and thrombocytopenia, with the shortest median time from treatment initiation to renal biopsy. Pathological analysis revealed diffuse global mesangiolysis (100%), a prominent GBM double contour, glomerular erythrocyte aggregation, and marked capillary contraction, with frequent acute tubulointerstitial lesions and interstitial inflammatory cell infiltration (Table 5, Figure 1K-L).

**Table 4.** Histopathologic characteristics in patients with anticancer drug-induced nephrotoxicity.

CTD (*n* = 25)		MTT (*n* = 22)		ICI (*n* = 5)	
Categories	n (%)	Categories	n (%)	Categories	n (%)
ATIN	12 (48)	TMA	16 (73)	ATIN	4 (80)
ACTIN	6 (24)	TMA+FSGS	2 (9)	ACTIN	1 (20)
TMA	3 (12)	MCD	1 (4)		
MCD	2 (8)	MN	1 (4)		
FSGS	2 (8)	ATIN	1 (4)		
		TMA+ATIN	1 (4)		

CTD: chemotherapy drugs; MTT: molecularly targeted therapies; ICI: immune checkpoint inhibitors; ATIN: acute tubulointerstitial nephritis; ACTIN: chronic tubulointerstitial nephritis with acute component; TMA: thrombotic microangiopathy; MCD: microscopic lesion nephropathy; FSGS: focal segmental glomerulonephritis; MN: membranous nephropathy.

**Table 5.** Clinicopathological characteristics of anticancer drug-induced thrombotic microangiopathy.

Variables	Anti-VEGF(R) (*n* = 11)	non-VEGFR- TKIs (*n* = 4)	Anti-VEGF+VEGFR-TKI (*n* = 4)	Gemcitabine (*n* = 3)
Time from Tumor Treatment to Renal Biopsy, months	10.0 (3.0–12.0)	4.0 (0.5–5.0)	1.1 (0.3–19.2)	0.8 (0.3–1.0)
Systolic Blood Pressure, mmHg	148.0 (131.0–158.0)	144.0 (138.0–155.0)	170.0 (152.0–187.0)	184.0 (165.0–190.0)
Diastolic Blood Pressure, mmHg	84.0 (72.0–101.0)	73.0 (60.0–85.0)	101.0 (93.0–116.0)	125.0 (115.0–130.0)
Hemoglobin, g/dL	112.0 (96.0–124.0)	87.0 (86.0–100.0)	127.0 (101.0–134.0)	70.0 (65.0–89.0)
Platelets, ×10^9^/L	158.0 (146.0–192.0)	189.0 (173.0–210.0)	145.0 (53.0–257.0)	76.0 (67.0–100.0)
LDH, U/L	276.0 (212.0–438.0)	193.0 (186.0–221.0)	300.0 (258.0–452.0)	559.0 (412.0–602.0)
Serum Creatinine, mg/dL	1.0 (0.8–1.6)	3.1 (0.6–3.9)	0.6 (0.5–0.7)	5.0 (2.1–5.2)
Serum Albumin, g/L	31.2 (28.3–34.1)	30.1 (27.9–32.7)	30.8 (24.0–35.2)	28.7 (23.4–31.8)
Proteinuria, g/24h	2.8 (2.1–4.0)	3.0 (0.9–3.3)	3.4 (1.1–7.2)	4.2 (3.0–5.6)
Microscopic Hematuria, /μl	14.1 (7.5–30.8)	180.8 (13.4–190.5)	1.9 (1.6–15.2)	67.5 (42.1–76.9)
Light Microscopy				
Ballooning of Capillary Collaterals, n (%)	11 (100)	1 (25)	1 (25)	1 (33)
Vitreous Alterations, n (%)	9 (81)	0	2 (50)	0
Double-Track Sign, n (%)				
Segmental	3 (27)	3 (75)	1 (25)	1 (33)
Global	7 (64)	1 (25)	3 (75)	2 (66)
Fibrin Thrombus in Loop, n (%)	4 (36)	1 (25)	1 (25)	1 (33)
Red Cell Aggregation in Loop, n (%)	5 (45)	3 (75)	1 (25)	2 (66)
Mesangial Lysis, n (%)				
Segmental	9 (81)	1 (25)	0	0
Global	2 (18)	0	4 (100)	3 (100)
Glomerular sclerosis, n (%)	9 (81)	2 (50)	2 (50)	1 (33)
Acute Tubulointerstitial Injury Score >1, n (%)	1 (9)	1 (25)	0	1 (33)
Chronic Tubulointerstitial Injury Score >1, n (%)	3 (27)	3 (75)	0	2 (66)
Arteriosclerosis, n (%)	7 (64)	3 (75)	4 (100)	2 (66)
Arterial TMA-Like Lesions, n (%)	0	1 (25)	0	0
Electron Microscopy				
Average Subendothelial Width, μm	1.6 (1.0–2.1)	0.9 (0.7–1.2)	2.0 (1.2–3.0)	2.7 (1.8–4.2)
Electron-Dense Deposits, n (%)	6 (54)	1 (25)	4 (100)	1 (33)
Podocyte Fusion ≥50%	5 (45)	1 (25)	1 (25)	0

Anti-VEGF(R): including anti-vascular endothelial growth factor (VEGF) drugs and vascular endothelial growth factor receptor (VEGFR)-tyrosine kinase inhibitors; non-VEGFR-TKIs: tyrosine kinase inhibitors that target other than VEGFR; aVEGF + VEGFR-TKI: anti-vascular endothelial growth factor drugs (Bevacizumab) combined with tyrosine kinase inhibitors (Anlotinib); LDH: lactate dehydrogenase.

In contrast, the MTT group exhibited a predominance of TMA (19/22 cases, 86%), with rare cases of MCD, IgAN, and ATIN (each 1 case, 4%). The histopathological features of TMA exhibited significant heterogeneity depending on the specific drug target. In the 11 cases of anti-VEGF(R)-related kidney injury, histopathological analysis revealed predominant lesions characterized by glomerular capillary ballooning (100%), exudative material (81%), mesangiolysis, subendothelial space widening, and hyaline droplet-like deposits in the subendothelial region, occasionally observed in capillary lumens. (Table 5, Figure 1C-E). In contrast, lesions associated with non-VEGFR-TKI treatment (*n* = 4) were milder, primarily characterized by endothelial injury, segmental glomerular mesangiolysis, and focal double contours of the GBM (Table 5, Figure 1F-G, H-I). Notably, one case of imatinib-induced TMA exhibited microvascular fibrinoid necrosis, red blood cell fragmentation, fibrous intimal thickening, endothelial foam cell degeneration, and arterial lumen occlusion (Figure 1J). These changes ultimately led to glomerular ischemia and contraction. Compared with anti-VEGF(R) monotherapy, the combination of anti-VEGF (bevacizumab) and VEGFR-TKI (anlotinib) therapies was associated with more severe hypertension, more rapid kidney injury, more extensive glomerular involvement (affecting >75% of glomeruli on average), and higher incidences of global mesangiolysis (100%) and diffuse basement membrane (GBM) double contours (75%) (Table 5).

**Figure 2.**
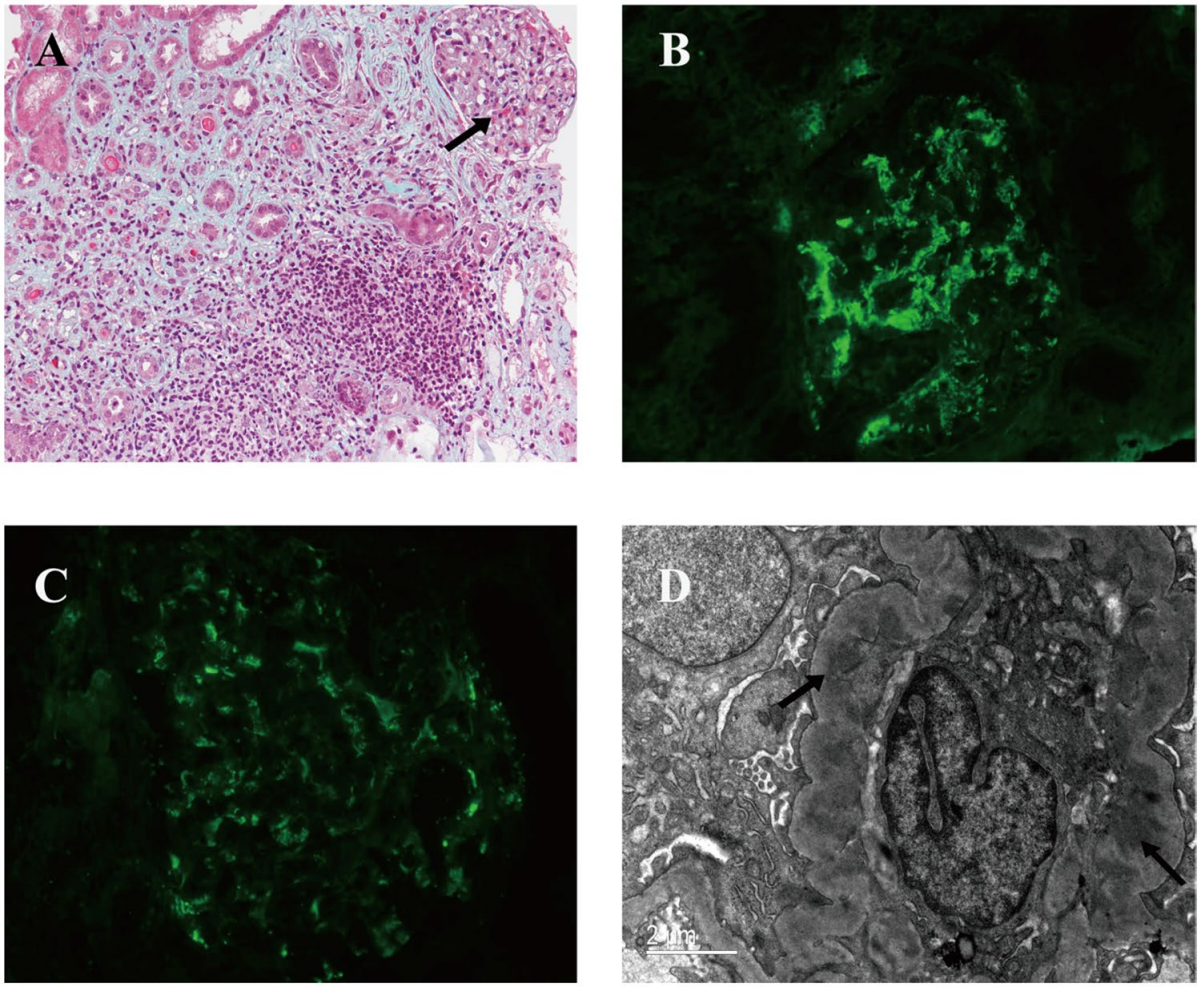
Two cases of ICI-induced ATIN complicated by IgA nephropathy, Masson stains (A) revealed eosinophilic material deposits within glomeruli, accompanied by increased interstitial inflammatory cell infiltration (arrows). There were IgA-dominant immune complex deposits (B, IgA immunofluorescence; original magnification, ×400) with C3 deposits along the capillary loops (C, C3 immunofluorescence; original magnification, ×400). Numerous mesangial electron-dense deposits are ultra-structurally indicated by arrows (D, electron microscopy).

Among patients treated with ICI, ATIN was identified as the predominant histopathological finding (3/5 cases, 60%), followed by chronic tubulointerstitial nephritis with acute component (1 case, 20%). Notably, 4 cases (80%) show IgA deposition on immunofluorescence (graded 1+ to 2+), with 2 cases demonstrating full diagnostic features of IgA nephropathy (mesangial IgA dominance with C3 co-deposition) (Table 6; Figure 2). Histological analysis revealed interstitial infiltrates predominantly composed of CD3+/CD4+ T lymphocytes and CD68+ macrophages (Supplementary Table 2; Supplementary Figure 1). Eosinophilic and plasma cells infiltration was observed in two cases (Figure 1A-B).

### Treatment and outcomes

The median follow-up duration was 23.0 months (9.0–45.0 months). In the CTD group (*n* = 25), patients with glomerular lesions (TMA/MCD/FSGS, *n* = 7) received the following interventions: 4 received RAAS inhibitors + corticosteroids, 2 required renal replacement therapy, and 1 received conservative therapy. During a median follow-up of 29.0 months (18.0–41.0 months), only 2 of these 7 patients (29%) achieved complete recovery (CR), while 3 (43%) progressed to ESKD. In contrast, patients with isolated TIN (*n* = 18) had a CR rate of 50% and no patients progressed to ESKD.

In the MTT group (*n* = 22), 11 patients discontinued anticancer treatment, 10 received RAAS inhibitors, 7 received corticosteroids and/or immunosuppressants, and 4 received conservative therapies. The median follow-up time was 19.0 months (9.0–36.0 months). Significant heterogeneity in renal recovery was observed based on drug targets and pathological subtypes: patients with anti-VEGF(R)-induced TMA (*n* = 11) had rapid renal recovery (median 0.5 months, IQR: 0.4–2.9 months), achieving overall remission of 90% (CR 45%; PR 55%). Proteinuria resolved completely in 73%. Among non-VEGFR-TKI-induced TMA cases (*n* = 4), three achieved PR while one progressed to ESKD due to imatinib-associated arterial TMA. Those receiving combination anti-VEGF + VEGFR-TKI therapy (*n* = 4) had a higher risk of severe hypertension and persistent proteinuria (PR 50%, NR 50%).

In the ICI group (*n* = 5), all patients received corticosteroids and/or immunosuppressants, while anticancer therapy was discontinued in one patient. The median follow-up time was 18.0 months (10.0–28.0 months). Notably, among the 3 patients with reduced serum complement C3, case 1 with ATIN+IgAN and reduced C3 died despite methylprednisolone therapy, case 3 with ATIN and reduced C3 achieved PR, and case 5 with ATIN+IgAN and reduced C3 showed NR. The remaining two patients with normal C3 levels had better outcomes: case 2 achieved CR following methylprednisolone pulse therapy, and case 4 showed PR.

**Figure 3.**
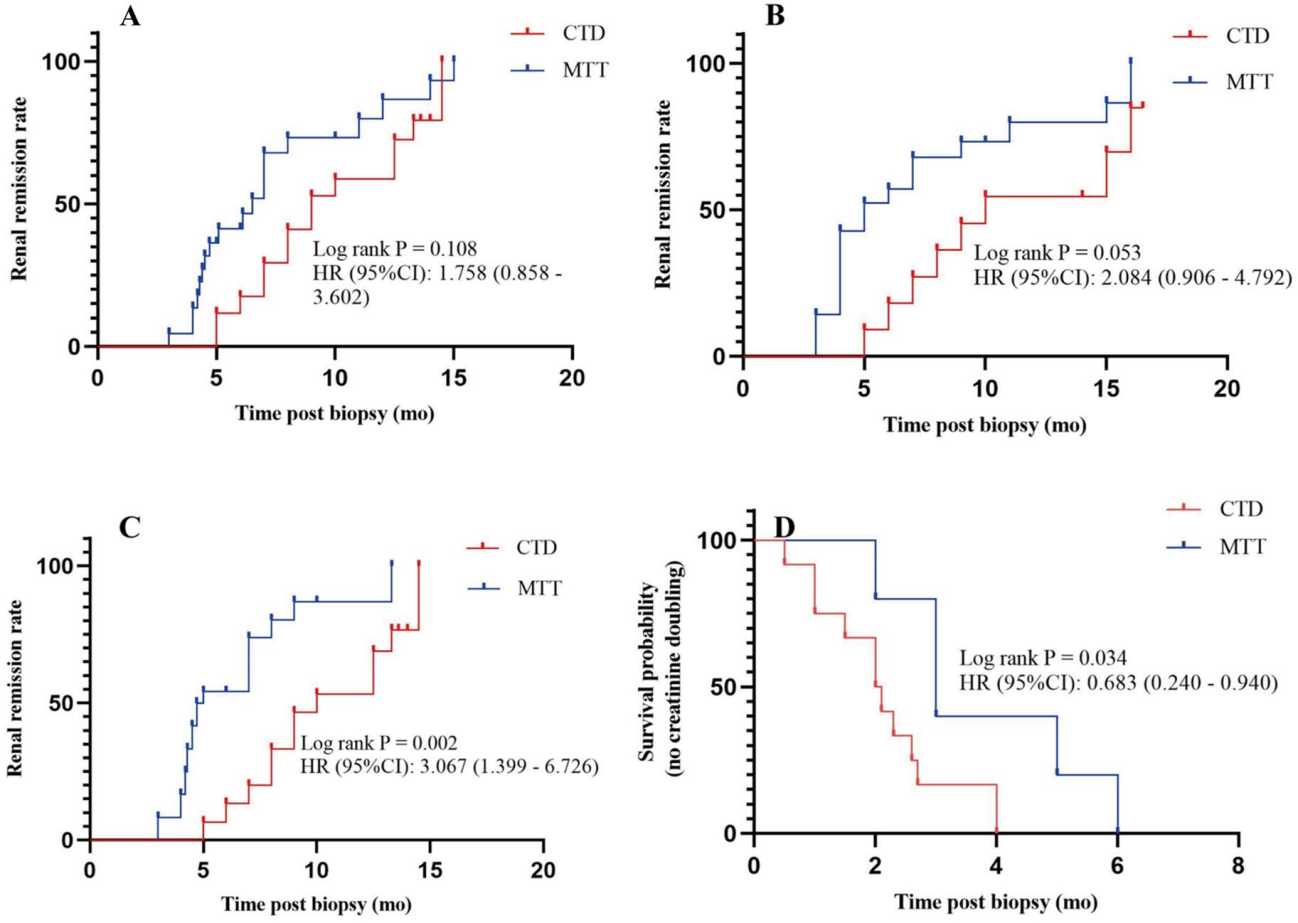
Kaplan-Meier analysis of renal outcomes in patients treated with CTD or MTT. (A) Renal remission rate in CTD and MTT groups. (B) Renal remission rate in patients with proteinuria induced by CTD or MTT. (C) Renal remission rate in patients with AKI induced by CTD or MTT. (D) Survival probability of patients with no creatinine doubling under CTD and MTT treatments. *P*-values were calculated by the log-rank test. CTD: chemotherapy drugs; MTT: molecularly targeted therapies.

**Figure 4.**
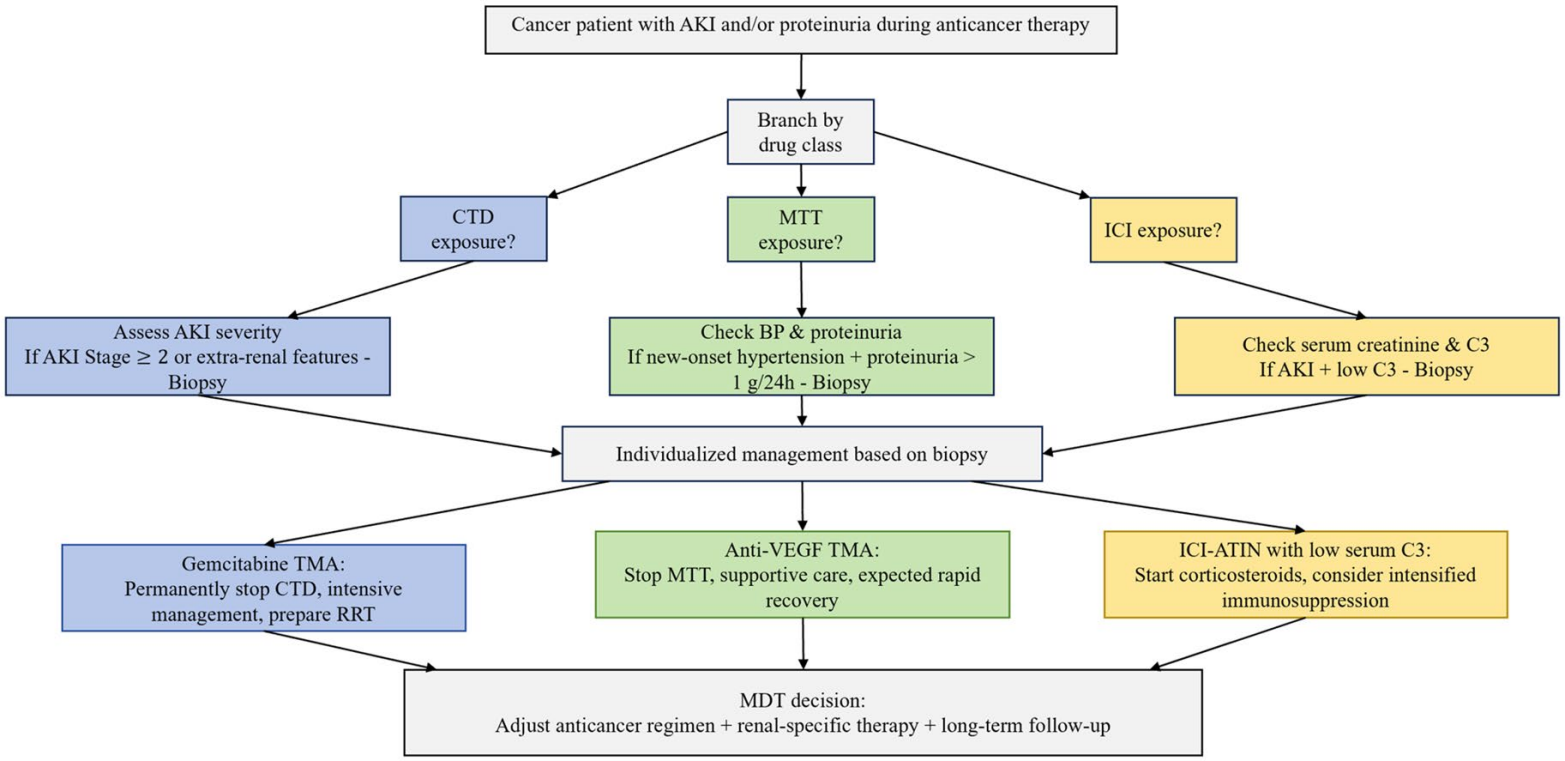
Clinical Algorithm for the Management of Anticancer Drug-Induced Nephrotoxicity. CTD: chemotherapy drugs; MTT: molecular targeted therapies; ICI: immune checkpoint inhibitors; AKI, acute kidney injury; BP: blood pressure; C3: complement C3; TMA: thrombotic microangiopathy; RRT: continuous Renal Replacement Therapy; Anti-VEGF: including anti-vascular endothelial growth factor (VEGF) drugs and vascular endothelial growth factor receptor (VEGFR)-tyrosine kinase inhibitors; ATIN: acute tubulointerstitial nephritis; MDT: multidisciplinary team consultation.

Survival curve analysis demonstrated no significant differences in overall renal survival rates between the CTD and MTT groups. Prognostic analysis was not performed in the ICI group owing to the insufficient sample size. Similarly, no significant differences in CR rates were observed among patients with proteinuria between the CTD and MTT groups (Figure 3A-B). However, among patients who developed AKI, the rate of renal remission was significantly higher in the MTT group than in the CTD group (hazard ratio [HR] = 3.067, 95% CI = 1.399 to 6.726; *p* = 0.002), with a median time to remission of 0.5 months versus 8.0 months (Figure 3C). Furthermore, the MTT group had a significantly lower risk of creatinine doubling than the CTD group (hazard ratio [HR] = 0.683, 95% CI = 0.240 to 0.940; Log-rank *p* = 0.034) (Figure 3D). Multivariable Cox regression analysis confirmed that MTT treatment remained independently associated with a lower risk of creatinine doubling after adjustment for tumor type, comorbidities, and other clinical factors (Supplementary Table 4). No significant difference was observed in median creatinine values between the CTD and MTT groups at the time of death (Figure 4).

## Discussion

This study comprehensively analyzed the clinicopathological characteristics of renal injuries induced by CTD, MTT, and ICI based on renal biopsy findings. CTD-associated nephrotoxicity presented with diverse pathological patterns, including notable glomerular lesions and a significant incidence of ATIN. In contrast, MTT-induced kidney injury predominantly presented as glomerular TMA, exhibiting marked pathological heterogeneity related to specific drug targets. Meanwhile, ICI-induced kidney injury primarily presented as ATIN, frequently accompanied by glomerular IgA deposition and reduced serum complement C3, highlighting the involvement of humoral immune perturbations.

It is noted that, except for ATIN, the glomerular lesions were not uncommon in the CTD group. The underlying pathogenic mechanism may involve different toxic effects that can target either renal tubular epithelial cells or podocytes, resulting in various observed pathological patterns [6–8]. These findings extend previous observations by highlighting that glomerular involvement is not uncommon in CTD-induced nephrotoxicity and may exacerbate AKI severity, warranting closer pathological evaluation of glomerular structures in clinical practice. In the comparison of pathological morphologies of MTT-related TMA, gemcitabine-induced TMA exerts direct cytotoxic effects on renal microvascular cells, likely through induction of oxidative stress and mitochondrial damage [9]. This mechanism is the basis for its unique pathological features of severe global mesangiolysis and diffuse GBM double contours. Clinically, this injury manifests as aggressive TMA frequently accompanied by significant hypertension and poor response to therapy, highlighting the irreversible nature of gemcitabine-induced nephrotoxicity [10–12]. We suggest that future studies validate specific endothelial injury biomarkers (e.g. soluble C5b-9 [13]) to better predict risks of these severe cases.

In stark contrast, MTT-induced kidney injury predominantly manifests as TMA, accounting for 86% of cases and establishing it as the hallmark pathology for this drug class. Critically, our analysis revealed marked heterogeneity in the histopathological manifestations of TMA, which is strongly correlated with the specific molecular target of the drug. VEGF blockade by anti-VEGF(R) therapies leads to endothelial nitric oxide synthase suppression, resulting in reduced nitric oxide and subsequent endothelial dysfunction. This mechanism underlies the characteristic glomerular capillary ballooning observed in these cases, which is consistent with functional endothelial impairment rather than structural necrosis [14,15]. These changes explain the rapid recovery of renal function following drug discontinuation. In contrast, non-VEGFR-TKIs may indirectly lead to endothelial nitric oxide suppression through the disruption of secondary signaling pathways [16], albeit to a lesser extent than direct VEGF blockade. This milder endothelial dysfunction typically results in the segmental glomerular lesions observed in these cases, which represent the mildest form of TMA. However, this level of dysfunction typically remains insufficient to cause widespread structural damage. A critical finding was that the BCR-ABL inhibitor imatinib was associated with severe arterial TMA leading to ESKD. This extends previous reports [17] and underscores that arterial involvement, though rare, suggests a poor renal prognosis in MTT-associated renal arteriolar thrombosis. Our study further emphasizes that the combination of anti-VEGF and VEGFR-TKI therapies results in synergistic toxicity [18–20]. This dual targeting amplifies endothelial damage, resulting in more extensive and severe TMA compared to single-agent inhibition. Identifying sensitive biomarkers for endothelial damage, such as those derived from urinary extracellular vesicles [21], could help in the early detection and monitoring of subtle functional impairment. The histopathological spectrum of MTT-TMA reflects distinct injury mechanisms driven by specific drug targets. Renal biopsy is therefore indispensable for recognizing this heterogeneity.

The predominant pathology in our ICI cohort was ATIN, consistent with current guidelines [22,23]. Beyond classic T-cell-mediated injury, we propose a broader mechanism by which ICI therapy may trigger humoral disturbances, leading to IgA autoimmunity and complement consumption. This is evidenced by glomerular IgA deposition in 80% of cases and reduced serum C3 levels in 60% of patients. Although IgA nephropathy following ICI has been reported [24], its frequent co-occurrence with complement reduction suggests a linked humoral dysregulation that is rarely seen in typical ATIN. This pattern suggests that alternative complement pathway activation may amplify kidney injury, a hypothesis further supported by reported cases of ICI-associated C3 glomerulopathy [25–27] (Supplementary Table 3). Therefore, monitoring serum C3 could help identify patients at risk of more severe injury, though this approach requires validation in larger prospective cohorts.

Despite these findings, the renal prognosis among all three groups was generally favorable, with the majority of patients achieving complete or partial recovery of renal function. Patients with MTT-associated AKI demonstrated the fastest renal remission compared to CTD. A study involving 1,459 patients following bevacizumab treatment revealed that only two patients had persistent proteinuria after discontinuation of the drug [28]. Conversely, a multicenter cross-sectional study on CTD-related AKI revealed a non-recovery rate of 48.3% among 258 patients [29].

Our biopsy-proven findings bridge current clinical management and the future of precision medicine in onco-nephrology [30]. For CTD, the wide range of pathologies (such as ATIN, TMA, and MCD) means that AKI should not be automatically attributed to acute tubular necrosis, and a kidney biopsy should be seriously considered. Gemcitabine-induced TMA is often irreversible and responds poorly to conventional therapies. Therefore, besides drug discontinuation, proactive measures and early prevention are crucial. For MTT, anti-VEGF(R)-related TMA is usually reversible and managed by stopping the drug, with no need for immunosuppressants. Additional interventions (e.g. immunosuppressants) should only be considered if kidney function fails to recover after drug cessation. In contrast, TMA associated with BCR-ABL inhibitors may involve arteries and has a poor prognosis. Additionally, combining anti-VEGF antibodies with VEGFR-TKI increases the severity of TMA, necessitating careful clinical monitoring. In our case of ICI injury, corticosteroid therapy proved effective, with frequent IgA deposition and reduced C3, indicating humoral immunity involvement. Consequently, assessing serum C3 levels may indicate disease activity.

Our research supports enhanced trial oversight and targeted drug labeling to better assess risk, consistent with recent oncology safety guidelines [31]. Specifically, the distinct nephrotoxic profiles we identified should guide clinical trial design, enhance drug labeling to indicate specific injury patterns and reversibility, and improve risk stratification strategies.

As a retrospective study, this research has several limitations. First, the potential influence of confounding factors, including metabolic or immune disorders related to the tumor itself, could not be eliminated. Second, the common use of combination therapies in cancer treatment makes it difficult to attribute kidney injury to a specific drug. Third, variable follow-up and cancer mortality may obscure long-term renal outcomes.

In conclusion, our findings demonstrate the distinct nephrotoxic profiles of major anticancer drug classes. CTD is associated with direct cytotoxicity and carries a high risk of irreversible injury. MTT drives functional TMA, which exhibits pathological heterogeneity dependent on the specific targets, and most cases can be rapidly reversed with drug withdrawal. ICI triggers immune dysregulation, involving both humoral disturbances and complement consumption. This clinicopathological knowledge is key to optimizing drug strategies for renal safety. Moreover, our findings highlight specific clinical red flags, such as hypertension with proteinuria in anti-VEGF TMA and AKI with low serum C3 in ICI-related immune-mediated nephritis. Therefore, our findings promote close collaboration between oncologists and nephrologists. This is essential for timely diagnosis and management, ensuring both renal and oncological outcomes.

## Supplementary Material

SUPPLEMENTARY MATERIAL.docx

## Data Availability

The data underlying this article will be shared upon reasonable request to the corresponding author.
