# Supplementary material for: Anticancer drug-induced nephrotoxicity: biopsy-proven patterns and outcomes across chemotherapy, targeted therapy, and immune checkpoint inhibitors
Source: Ren Fail. 2025 Nov 25;47(1):2590283. doi: 10.1080/0886022X.2025.2590283 (PMC12649773; doi:10.1080/0886022X.2025.2590283)
Supplement: SUPPLEMENTARY MATERIAL.docx [file IRNF_A_2590283_SM6920.docx]

**SUPPLEMENTARY MATERIAL**

**File list**

1. Supplementary Table 1.

2. Supplementary Table 2.

3. Supplementary Table 3.

4. Supplementary Figure 1.

| **Supplementary Table 1. Clinicopathological Characteristics and Outcomes in a Cohort of Patients with Anticancer Drug-Associated Kidney Injury** | | | | | |
| --- | --- | --- | --- | --- | --- |
| No. | Anticancer Regimen | Clinical Presentation | Treatment | Pathological Presentation | Renal Outcome |
| 1 | AC-T | AKI | Discontinuation+Steroids+ACEI/ARB | ACTIN | CR |
| 2 | TP | AKI | Steroids | ATIN | CR |
| 3 | TP | AKI | Steroids | ATIN | PR |
| 4 | TP | AKI | Steroids | ATIN | CR |
| 5 | 5-FU | AKI | ACEI/ARB | ATIN | CR |
| 6 | Pemetrexed+Pt | AKI, PRO | Steroids | ACTIN | NR |
| 7 | CCNU+DDP+BRAF | AKI, PRO | Steroids+ACEI/ARB | ACTIN | PR |
| 8 | Pemetrexed+Pt | AKI, PRO | Dialysis | ATIN | PR |
| 9 | TP | AKI, PRO | Steroids+ACEI/ARB | ATIN | CR |
| 10 | CAPOX | AKI, PRO | Steroids | ATIN | CR |
| 11 | 5-FU | AKI, PRO | Steroids+ACEI/ARB | FSGS | CR |
| 12 | CF | AKI, PRO | ACEI/ARB | ATIN | NR |
| 13 | EP | AKI, PRO | Steroids | ACTIN | PR |
| 14 | GEM+T | AKI, PRO | Dialysis | TMA | ESKD |
| 15 | ADT | AKI, PRO | Steroids | ATIN | PR |
| 16 | TP | AKI, PRO | Dialysis | ACTIN | Dialysis |
| 17 | 5-FU | AKI, PRO | None | ATIN | CR |
| 18 | GEM | AKI, PRO | Steroids+ACEI/ARB | TMA | PR |
| 19 | CAPOX | AKI, PRO | Conservative therapy | ACTIN | PR |
| 20 | GEM | AKI, PRO | Steroids+ACEI/ARB | TMA | ESKD |
| 21 | TP | PRO | Dialysis | FSGS | ESKD |
| 22 | 5-FU | PRO | Steroids+ACEI/ARB | ATIN | CR |
| 23 | TP | PRO | Steroids+ACEI/ARB | MCD | CR |
| 24 | 5-FU | PRO | Steroids+ACEI/ARB | ATIN | CR |
| 25 | SP | PRO | Conservative therapy | MCD | PR |
| 26 | Bevacizumab + Pemetrexed+Pt | AKI, PRO | Conservative therapy | TMA+FSGS | PR |
| 27 | Bevacizumab+5-FU | AKI, PRO | ACEI/ARB | TMA | CR |
| 28 | Bevacizumab+IRI + OXA + RAL | AKI, PRO | Discontinuation+Conservative therapy | TMA | PR |
| 29 | Bevacizumab | PRO | ACEI/ARB | TMA | Dead |
| 30 | Bevacizumab | PRO | Discontinuation+ACEI/ARB | TMA | CR |
| 31 | Bevacizumab | PRO | Steroids | TMA | CR |
| 32 | Bevacizumab+anlotinib+ Pemetrexed+Pt | AKI, PRO | Steroids | TMA | CR |
| 33 | Bevacizumab+anlotinib+TP | AKI, PRO | Discontinuation | TMA | PR |
| 34 | Bevacizumab+anlotinib+5-FU | AKI, PRO | Discontinuation+Steroids | TMA | PR |
| 35 | Lenvatinib | PRO | Discontinuation+ACEI/ARB | TMA | Dead |
| 36 | Lenvatinib +5-FU | PRO | ACEI/ARB | TMA+FSGS | PR |
| 37 | Apatinib+ CF | AKI, PRO | Discontinuation+Conservative therapy | TMA+ATIN | PR |
| 38 | Apatinib+5-FU | PRO | Discontinuation+Conservative therapy | MCD | CR |
| 39 | Sorafenib | PRO | Discontinuation+ACEI/ARB | TMA | CR |
| 40 | Fruquintinib | PRO | Discontinuation+ACEI/ARB | TMA | CR |
| 41 | Sunitinib | PRO | ACEI/ARB | TMA | PR |
| 42 | Dasatinib+ CF | AKI, PRO | Steroids | TMA | PR |
| 43 | Dasatinib+Camrelizumab | PRO | Discontinuation+ACEI/ARB | TMA | PR |
| 44 | Nilotinib | PRO | Discontinuation+Steroids | MN | CR |
| 45 | Imatinib | AKI | Steroids | TMA | Dialysis |
| 46 | Aumolertinib | AKI, PRO | Steroids | ATIN | PR |
| 47 | Cetuximab | PRO | ACEI/ARB | TMA # | PR |
| 48 | Camrelizumab（anti-PD-1） | AKI | Discontinuation+Steroids | ATIN | CR |
| 49 | Camrelizumab（anti-PD-1） | AKI, PRO | Steroids | ATIN+IgAN | NR |
| 50 | Sintilimab（anti-PD-1） | AKI, PRO | Steroids | ATIN+IgAN | Dead |
| 51 | Sintilimab（anti-PD-1） | AKI, PRO | Steroids | ACTIN | PR |
| 52 | Nivolumab（anti-PD-1） | AKI | Steroids | ATIN | PR |

AC-T: Doxorubicin+Cyclophosphamide+Paclitaxel; TP: Docetaxel/Paclitaxel+Cisplatin/Carboplatin; 5-FU: Capecitabine/Tegafu; CCNU+DDP+BRAF: Fotemustine+Cisplatin+Dabrafenib; Pemetrexed+Pt: Pemetrexed+ Nedaplatin/Carboplatin; CAPOX: Capecitabine+Oxaliplatin; CF: Car #boplatin+Fluorouracil; FOLFOX: Fluorouracil+Oxaliplatin; EP: Etoposide+Cisplatin; GEM+T: Gemcitabine+Paclitaxel; ADT: Bicalutamide+Goserelin; IRI + OXA + RAL: Irinotecan+Oxaliplatin+Raltitrexed; Discontinuation: Discontinue drug; ACEI/ARB: Renin-Angiotensin-Aldosterone System inhibitors; AKI: acute kidney injury; PRO: proteinuria; ATIN: acute tubulointerstitial nephritis; ACTN: acute combined chronic tubulointerstitial nephritis; FSGS: focal segmental glomerulosclerosis; TMA: thrombotic microangiopathy; MCD; minimal change disease; MN: membranous nephropathy; IgAN: IgA nephropathy; CR: complete recovery of serum creatinine; PR: partial recovery of serum creatinine; NR: non-remission is the failure to achieve CR or PR despite clinical therapy; ESKD: End-Stage Kidney Disease; TMA #: arterial TMA.

**Supplementary Table 2. Clinicopathological Characteristics of 5 Cases of ICI-induced Nephrotoxicity**

| **No.** | **1** | **2** | **3** | **4** | **5** |
| --- | --- | --- | --- | --- | --- |
| Age, years | 61 | 56 | 45 | 65 | 56 |
| Gender | Male | Male | Male | Male | Male |
| ICIs | Sintilimab | Camrelizumab | Nivolumab | Sintilimab | Camrelizumab |
| Immunohistochemistry |  |  |  |  |  |
| CD3 | NA | ++ | ++ | ++ | ++ |
| CD4 | NA | ++ | ++ | ++ | ++ |
| CD8 | NA | ++ | + | ++ | + |
| CD68 | NA | NA | ++ | ++ | + |
| CD138 | NA | NA | + | ++ | ＋ |

**Supplementary Table 3：Summary of reported cases of serum C3 decline associated with immune checkpoint inhibitors.**

|  | Author | Age.yrs | Sex | Cancer | ICI(s) received | Course(s) of ICI(s) | Clinical  Manifestation（s） | sC3 (g/L) | sC4 (g/L) | TIN | pathological manifestations | Treatment | Outcom |
| --- | --- | --- | --- | --- | --- | --- | --- | --- | --- | --- | --- | --- | --- |
| 1 | Simon Ville[1] | 73 | M | NSCLC | Pembrolizumab | 3 cycles | AKI+nephrotic syndrome | 0.26 g/L↓ | Normal | None | C3 GN | Prednisone 60mg/d | PR |
| 2 | Zhi Yang[2] | 68 | M | NSCLC | Pembrolizumab | 19 cycles | AKI+nephrotic syndrome+gross hematuria | 0.558g/L↓ | 0.123g/L↓ | ATIN | C3 GN, C3 (2+) | Prednisone 60mg/d+CTX+dialysis | Dialysis |
| 3 | Tarek Ashour[3] | 68 | M | malignant melanoma | Pembrolizumab+Nivolumab | 6 cycles | AKI+gross hematuria+Proteinuria | 79 mg/dl↓ | Normal | None | C3 GN, C3 (3+), C1q (1+) | Prednisone 60mg/d | PR |
| 4 | Tao Zhao[4] | 73 | M | SCLC | Durvalumab | 6 months | AKI+gross hematuria+Proteinuria | 0.478 g/L↓ | NA | ATIN | IgAN | Methylprednisolone pulses+RTX | Dialysis |
| 5 | De-Hu Li[5] | 74 | M | SCLC | Durvalumab | 10 cycles | Swelling and itching of the skin | ↓ | NA | None | diffuse cutaneous systemic sclerosis | Prednisone 40mg/d+MMF | Skin was hard, and dysphagia persisted |
| 6 | Na Hong[6] | 57 | M | Hepatocellular carcinoma | Tirelizumab | 5 cycles | bloody diarrhea | ↓ | ↓ | None | colitis | Methylprednisolone 40mg/d | Gastrointestinal bleeding stopped |

M: Male; ICI: Immune checkpoint inhibitor; AKI: Acute kidney injury; sC3: Serum C3 level; sC4: Serum C4 level; TIN: Tubular-interstitial injury; ATIN: Acute tubular-interstitial injury; C3 GN: C3 Glomerulonephritis; IgAN: IgA Nephropathy; CTX: Cyclophosphamide; RTX: Rituximab; MMF: Mycophenolate Mofetil; PR: Partial remission; NA: Not acquired.

**Supplementary Table 4. Risk Factors for Creatinine Doubling Secondary to Anticancer Drugs**

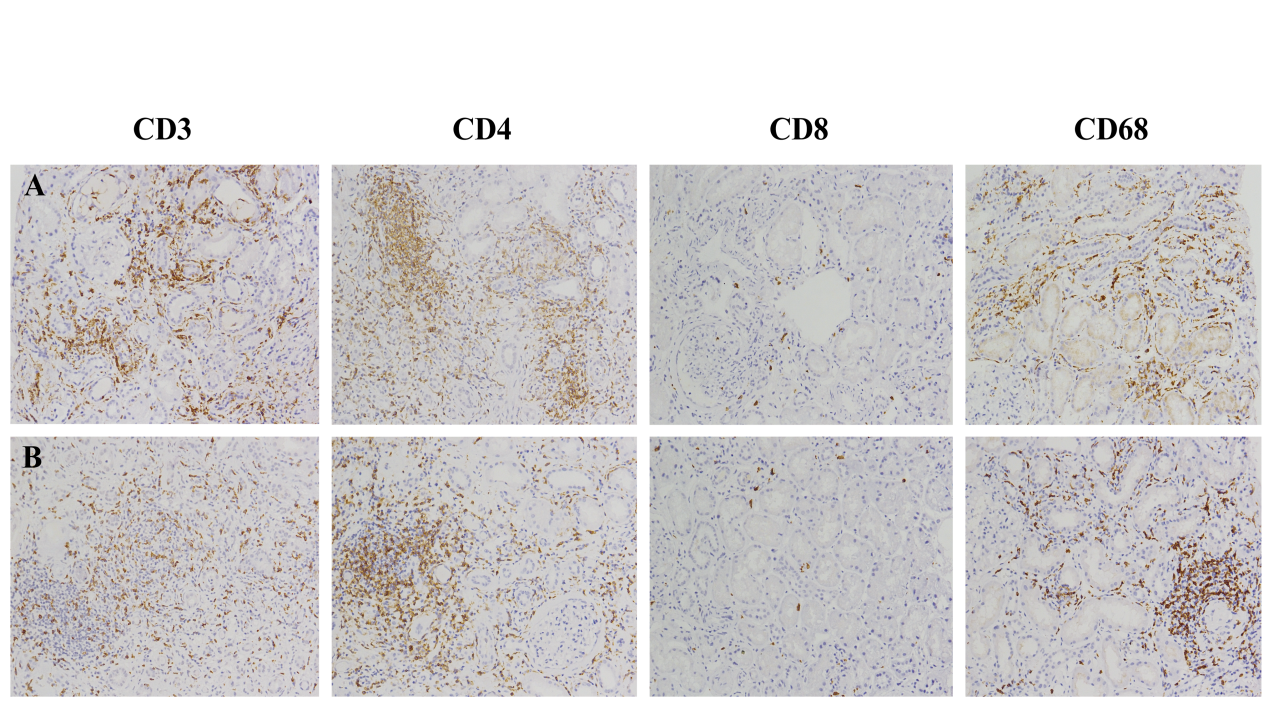


**Supplementary Figure 1.** Immunohistochemistry of renal biopsies from immune checkpoint inhibitors. Renal biopsies from (A) patient 2 and (B) patient 3 reveal an inflammatory infiltrate composed of CD3+/CD4+ T lymphocytes and CD68+ macrophages. Scale bar, 200 mm.

References

[1] S. Ville, C. Kandel-Aznar, V. Frémeaux-Bacchi, F. Fakhouri, C3 glomerulonephritis in a patient treated with anti-PD-1 antibody, Eur J Cancer 125 (2020) 46-48.

[2] Z. Yang, H. Xu, S. Gou, H. Wu, Z. Hu, Pembrolizumab induced-C3 glomerulonephritis and RBC cast nephropathy: a case report, BMC Nephrol 24(1) (2023) 145.

[3] T. Ashour, G. Nakhoul, P. Patil, P. Funchain, L. Herlitz, Immune Check Point Inhibitor-Associated Glomerulonephritis, Kidney Int Rep 4(2) (2019) 355-359.

[4] T. Zhao, X. Zhang, Y. Li, T. Su, Durvalumab-Associated Crescentic Glomerulonephritis With IgA Vasculitis-Like Features, Kidney Int Rep 10(7) (2025) 2489-2490.

[5] D.H. Li, X.Z. Xiong, Immune Checkpoint Inhibitor-Associated Systemic Sclerosis in the Treatment of a Small Cell Lung Cancer Patient with Durvalumab: A Case Report, Clin Cosmet Investig Dermatol 17 (2024) 663-669.

[6] N. Hong, B. Wang, H.C. Zhou, Z.X. Wu, H.Y. Fang, G.Q. Song, Y. Yu, Multidisciplinary management of ulcerative colitis complicated by immune checkpoint inhibitor-associated colitis with life-threatening gastrointestinal hemorrhage: A case report, World J Gastrointest Surg 16(7) (2024) 2329-2336.
